# The discursive construction of corporate identity in the corporate social responsibility reports: A case study of Starbucks

**DOI:** 10.3389/fpsyg.2022.940541

**Published:** 2022-08-24

**Authors:** Xuyan Li

**Affiliations:** School of English for International Business, Guangdong University of Foreign Studies, Guangzhou, Guangdong, China

**Keywords:** CSR reports, corporate identity, discursive construction, discourse-historical approach, critical discourse studies, corporate discourse, CSR communication

## Abstract

Both corporate identity and corporate social responsibilities are of strategic importance to companies’ reputation and competitiveness. From a social constructivist view, identity is constructed in discourse. Therefore, this study sets out to investigate how corporate identity is discursively constructed in corporate CSR communication. Taking Starbucks as an example, this corpus-assisted study explores how Starbucks deploys nomination, predication, and intensification strategies and the corresponding linguistic resources to discursively construct itself and its main stakeholder groups in the CSR reports from the perspective of Discourse-Historical Approach to Critical Discourse Analysis. Also, how Starbucks addresses or presents issues in which scandals or problems reside is investigated. The findings show that Starbucks explicitly constructs itself as the supportive care-taker of the partners, faithful deliverer of good customer experience, powerful helper of poor farmers, and CSR-conscious selector of suppliers, who takes a strongly committed and proactive CSR stance through the discourse. However, behind such discursive construction are the hidden ideologies and corporate agenda of a capitalistic nature, with Starbucks veiling the power dominance and unequal power relations. This study not only contributes to the understanding of the discursive construction of corporate identity, but also helps raise peoples’ awareness of the power game at play behind the corporate discourse.

## Introduction

The word identity is rooted in the Latin attribute idem, which means ‘the same,’ and later nominalized as identitas, meaning sameness. It is used to designate the particular characteristics by which a person or an entity becomes recognizable (Bamberg and Dege, 2021). Once a huge philosophical problem explored passionately by philosophers, the concept of identity has grown to be extensively investigated in various academic disciplines like sociology, psychology, and anthropology. Then, with the emergence and proliferation of companies, the concept of identity has also been applied to companies/organizations by scholars and practitioners in the business and management fields, where companies are metaphorically deemed as actors who think, reason and behave, capable of conceiving of themselves and others as having identities (Brunsson, 1989; Sevón, 1996). By nature, corporate identity answers the questions ‘who we are as a company,’ ‘what we stand for,’ ‘what we do,’ ‘how we do it,’ and ‘where it is going’ (Bernstein, 1984; Albert and Whetten, 1985; Melewar and Jenkins, 2002), which can serve to differentiate one company from the others. Research (as cited in Simões et al., 2005) has proved the existence of positive correlations between a positive corporate identity of a company to superior performance. Therefore, companies are motivated to construct an identity favorable to the stakeholders (e.g., customers and investors), so as to attract and maintain them in the hope of securing and improving their financial performance.

To construct a favorable corporate identity, companies have begun to devote themselves to an ever-growing trend in the business practices, that is, corporate social responsibility (hereafter CSR). Stimulated by the deterioration of the environment and the proliferation of social problems, peoples’ environmental and social consciousness has become all-time awakened. Therefore, more than ever before, companies are under enormous pressure and scrutiny from various stakeholder groups, including but not limited to governments, NGOs, interest groups (e.g., environmentalists), investors, and consumers. Companies are deemed as corporate citizens responsible for the sustainability of not only the economy, but also the society and the planet earth. Indeed, research has found that consumers attach great importance to a company’s CSR practices (e.g., Vătămănescu et al., 2021), providing further incentives for companies to pursue CSR and construct a favorable identity in this regard. In such contexts, a good corporate identity necessarily involves the active fulfillment of CSR and the proactive communication of CSR efforts in an effective way to the stakeholders (Kotler, 2011; Tata and Prasad, 2015; Liu and Komal, 2022).

In terms of the construction of identity, research in various fields like anthropology, linguistics, sociology, history, and psychology, to name a few, has firmly established and acknowledged the essential role of linguistic strategies and discourse processes in the construction and negotiation of identities (De Fina et al., 2006). In this light, identity is viewed from the social constructionist perspective (see, e.g., Berger and Luckmann, 1966) and discourse perspective (see, e.g., Fairclough, 1989). That is, identity is not something static and fixed, but constantly being socially constructed, maintained, and negotiated, through discourse and communication (e.g., Benwell and Stokoe, 2006; De Fina, 2010). As such, corporate identity can be construed as a process, something companies ‘do’ or ‘perform,’ rather than a static attribute that they ‘possess’ (Bucholtz and Hall, 2005; Bamberg et al., 2011). In this sense, companies construct their identities in their communication to the stakeholders, and this is achieved by the use of discourse. Following the social constructionism and discourse perspectives, discourse is both socially constituted and constitutive, which is a social practice that is both socially conditioned and consequential (Fairclough, 1989). Besides, discourse can construct and maintain or challenge general worldviews, or rather, ideologies, as “ideologies may be enacted in ways of interaction (and therefore in genres) and inculcated in ways of being identities (Fairclough, 2003, p: 218).” Therefore, the discursive construction of corporate identity does not merely construct certain identities, but also constructs and convey certain worldviews or ideologies. If unaware of such hidden ideologies, people may take these worldviews for granted and buy into them without questioning whether other alternatives exist.

In this study, corporate identity is considered to be discursively constructed in the corporate communication with hidden ideologies at play. In particular, the corporate identity in the CSR dimension is discursively constructed in the company’s annual CSR reports, whose aim in disclosing the company’s CSR information is to construct a positive identity, manage the corporate image and engage in dialogs with the stakeholders (Perrini, 2005).

Therefore, it is this study’s objective to investigate how a company use discourse strategies and linguistic resources to construct its identity in the CSR reports and to reveal the hidden corporate agenda behind.

## The case of Starbucks

This study intends to conduct and present a case study of Starbucks. The reasons are manifold. First, seen in the restaurant industry or coffeehouse industry, or as a combination of both, Starbucks comparatively stands out and is unquestionably world-renowned. Second, it has enjoyed a high CSR profile in its own industry and has appeared on many CSR-related ranking lists, which can more or less affirm its CSR achievements. Third, it is one of the pioneers in publishing annual CSR reports, starting as early as 2001, which guarantees a relatively sizable corpus for analysis.

### Starbucks’ CSR reputation and stance

As a global coffee and food retailer, Starbucks has established stores in more than 80 countries and regions. In terms of CSR reputation, it has ranked eighth in Fortune’s ranking of the Most Admired Companies 2022, and has remained one of the top 10 in this ranking for many years. One of the attributes of reputation this ranking evaluates is social responsibility to the community and the environment. In addition to this, Starbucks is on the Dow Jones Sustainability Index and included in many other Environment, Society, and Governance-related lists, such as Barron’s 100 Most Sustainable US Companies, Forbes’ America’s Best Large Employers, and Sustainalytics’ Global Sustainability Index. Moreover, it is ranked on Ethisphere’s World’s Most Ethical Companies list for 12 years in a row. All these official rankings demonstrate that Starbucks’ CSR efforts are world-widely acknowledged, rendering a relatively positive corporate identity in terms of CSR.

In its own industry, it is also perceived to be taking the lead in CSR. According to Restaurant Business magazine (Brooks, 2009), consumers identity Starbucks as one of the few green and socially responsible companies among all restaurant chains.

However, despite the high ranking in terms of CSR and the consumers’ impression of its greenness, Starbucks also has been criticized for some of its CSR practices. In particular, its tax avoidance in the United Kingdom (Campbell and Helleloid, 2016), the alleged racial bias of its staff (Karlsen and Scott, 2019), and its attitudes toward unionization (Morrow, 2022) have attracted strong criticism, leading to varying degrees of identity crises and damage to its brand image. Since CSR reports are important venues for the company’s communication to the external audience on the CSR issues, it would be expected that Starbucks made some responses to the criticisms it received in its CSR reports. Whether or not these responses are present, and how are they made will be revealing of Starbucks’ deliberate attempts of identity construction.

### Starbucks’ CSR reporting

The most important vehicle for corporate communication on its CSR performance and plans is the annual CSR report. In terms of corporate reporting on CSR, Starbucks issued its first official CSR report as early as in 2001, making it one of the pioneers who provided a stand-alone annual corporate report specifically focused on CSR. Moreover, along with each report, there is also an independent assurance report from Moss Adams, serving as an endorsement and external audit on Starbucks’ CSR disclosure in the report.

### CSR in the coffee industry and restaurant industry

To examine Starbucks’ discursive construction of its corporate identity in the CSR dimension, it would not be as informative to disregard the industry context. With regards to Starbucks’ products and services, it can be identified as belonging to the coffee industry as well as the restaurant industry. A briefing on the CSR concerns of these two industries would be insightful for the analysis of Starbucks’ identity construction in its CSR reports.

Nowadays, the coffee chain is buyer-driven, as international traders, retailers and major coffee roasters become powerful actors in the coffee chain (Bitzer et al., 2008). As a consequence, many coffee producers have been pushed down below the poverty line, sometimes even to starvation (Muradian and Pelupessy, 2005). The constant pressure of unstable income and the lack of regulation and enforcement mechanisms for the provision of public goods result in sustainability challenges at the production level, e.g., poor working condition, biodiversity decline and environmental degradation (Bitzer et al., 2008). Furthermore, as farmers are not well-instructed to grow coffee beans in an efficient manner, or informed about marketing advantages and the quality demand on the international market (Bitzer et al., 2008), they are further disadvantaged.

Besides the challenges placed on the farmers, the coffee industry also faces sustainability challenges regarding the environment, e.g., the harmful production practices and the ‘technification’ of coffee cultivation which has a negative effect on the local fauna (Rice, 2003). The coffee industry is, by nature, unsustainable, as coffee farming leads to vulnerability to tropical soil erosion and leaves substantial water footprint. Since the 1990s, the coffee industry has embraced new consumption patterns which showed a growing interest in specialty, fair-traded, and organic coffees (Ponte, 2002).

Within the restaurant industry, environmental issues and green awareness have attracted growing interest, with more and more consumers becoming environmentally concerned and ecologically conscious about their choices (Hu et al., 2010). Following the sustainability trend, companies in the restaurant industry have embraced green practices of developing products and services respectful to the environment, energy conservation, water efficiency, recycling and so on (Tan and Yeap, 2012; Jang et al., 2015).

From what has been provided, the most important stakeholder groups that are affected by the production of coffee are the environment and the farmers, and the consumer trend for coffee industry as well as restaurant industry is ethical consumption and green practices. Responding to these growing trends, Starbucks has been working closely with coffee farmers and promoting sustainability through reusable cups, recycles, LEED-certified building and other environmentally friendly practices (Ruzich, 2008). Despite these efforts, some CSR practices of Starbucks have been found to be not as environmentally responsible as Starbucks claimed to be. For instance, although fair trade coffee is offered as an option, it is brewed in Starbucks stores only once a month (Ruzich, 2008).

In this light, some green practices of companies have been doubted and have been regarded as a kind of green marketing (see as cited in Tsai et al., 2020). Therefore, the discursive construction of corporate identity in the CSR reports warrants a critical perspective. As critical discourse studies (hereafter, CDS) do not stop at the investigation of linguistic resources and discursive strategies for constructing corporate identity, but it also attempts to uncover the hidden values and ideologies behind the use of discourse.

As such, it would be insightful to investigate how Starbucks, as a coffee giant, addresses these stakeholder concerns in its discursive construction of the corporate identity in CSR reports from a CDS perspective.

## Critical discourse studies on corporate identity construction

Among the existing literature, studies taking a CDS perspective, or a linguistic approach on corporate identity are still rather limited in number. Nevertheless, such studies shed light on what a CDS perspective can reveal in the discursive construction of the corporate identity in companies’ CSR communication.

For instance, Shinkle and Spencer (2012) conduct a critical discourse analysis on the CSR reports of multinational automotive companies and show how the companies deploy rhetorical resources to strategically position themselves in the global marketplace as global corporate citizens. They discover that “value talk” pervades these CSR reports, serving as a rhetorical resource for constructing an identity of legitimacy.

Another informing study is conducted by Livesey (2001), who critically analyzes the eco-discourse produced by Royal Dutch/Shell Group, and finds that green rhetoric is used for sensemaking. She concludes that the construction of green identity reflects the importance of green ideologies for companies’ competitive survival, and contends that the analysis of the corporate eco-discourse can help reveal how people’s perception of the CSR are shaped. While this study has a solid theoretical framework and takes a critical perspective, it focuses specifically on the discourse level, treating discourse as the unit for analysis and does not include the investigation of linguistic resources and discursive strategies.

Similarly, Chen and Eriksson (2019) examine 22 corporate stories of healthy snack companies on their corporate websites, and find that companies use moral discourse of healthy eating to represent themselves as producers of healthy food through communication strategies used to persuade consumption. To be more specific, they report that these companies use othering discourse to distinguish themselves from the big corporate world, presenting themselves on the good side as humble, ordinary, struggling companies while placing the profit-driven mega food companies on the other side as villains. In this sense, they argue that the construction of a conscious, eco, green, and sustainable corporate identity serves a profit-driving capitalistic end.

To conclude, studies taking a CDS perspective to analyze the discursive construction of the corporate identity in CSR communication confirm the importance of the critical perspective and discourse analysis on identity construction. However, they mainly rely on the close-reading of texts, which may lack the quantitative insights on the use of linguistic resources for different discursive strategies.

## Theoretical foundation

### Linking social constructionism and corporate identity

The notion of social constructionism is popularized by Berger and Luckmann (1967), who observed that all knowledge is derived from and maintained by social interactions, on the basic of which reality is socially constructed. In other words, social reality can be seen as being constructed through a system of socio-cultural and interpersonal interactions in people’s everyday life. According to them, the construction of reality happens through three levels of processes: externalization, objectivation, and internalization (Berger and Luckmann, 1966). People use discourse to describe and interpret reality, which in turn becomes artifacts or practices. Then, these discourses enter into the social world, being exchanged, and reproduced by other people, which then become an object of consciousness for people and turn into a kind of factual existence of truth. Finally, people internalize the constructed truth or make it part of the everyday practices, which then maintains the constructed reality (Burr, 1995). In this sense, reality is both subjectively and objectively constructed (Segre, 2016). And important for the construction is communication, argued by Berger and Luckmann (1967), who contend that ongoing communication of stable or changed actions produces reality.

Since the 1980s, the concept of corporate identity has gained pivotal position in the realm of organizational studies. Since the publication of Albert and Whetten (1985) seminal text, interest in organizational/corporate identity has thrived, giving birth to a plethora of studies on corporate identity from management and communication perspective. While the importance of this concept is widely recognized, there has been a lack of consensus on its definitions (Balmer, 1995). According to Balmer (1995), the existing approaches to conceptualizing corporate identity can be identified as constituting sever distinct schools of thought, e.g., the total corporate communication and visual communication school, etc. With the diverse perspectives and the corresponding diverse definitions on this concept, it can be said that the concept of corporate identity is “suffering an identity crisis” (Whetten, 2006, p: 220).

In this study, a social constructionist perspective on corporate identity is adopted, which views corporate identity as the socially constructed products of relationships between the company and its stakeholders regarding “who the company is” (Corley et al., 2006), deriving from a complex of interactions by different actors from different professional groups and hierarchical levels (Harrison, 2000). To be more specific, companies pursue various corporate activities aimed at constructing its identity ongoingly, the repetition of which generates meaning over time. And companies have the sovereignty to take control of these activities to spell out what they stand for and where they are heading. Then, companies communicate to the internal and external stakeholders through discourse about their corporate activities and values that define who they are (Otubanjo et al., 2008). Here, an emphasis is put on the company’s ongoing communication to the stakeholders in terms of identity construction.

### Critical discourse studies and its discourse-historical approach

Following the social constructionist perspective on corporate identity, this study adopts CDS as the research paradigm for investigating the discursive construction of corporate identity in Starbucks’ CSR reports.

In this study, the term CDS is used in place of CDA (Critical Discourse Analysis) following the recommendation in the edited book of Wodak and Meyer (2016). In this book, it is contended that CDA is not a method of doing critical discourse analysis, as there is not ‘a’ method of CDA, but many, depending on the analyst’s aims, expertise, time, critical goals, and the research project. And the heterogeneity of methodological and theoretical approaches shows that CDS ‘are at most a shared perspective on doing linguistic, semiotic or discourse analysis’ (van Dijk, 1993, p: 131). Therefore, he recommends to use Critical Discourse Studies for the theories, methods, analyses, and other practices in conducting a critical study on discourse. This proposal is taken seriously by other influential scholars in the CDS domain (Wodak and Meyer, 2016).

CDS views the use of language as a ‘social practice’ that is both determined by social structure and, at the same time, contributes to stabilizing and changing that structure (Fairclough and Wodak, 1997). Therefore, it aims to shed light on how discourse functions in constituting and disseminating knowledge, and in organizing social institutions and exercising power, so as to enable human ‘enlightenment and emancipation’ (Wodak and Meyer, 2016). Concepts central for CDS are ideology, power, and discourse.

Two key concepts are integral to CDS, namely, ideology and power. To begin with, ideologies in CDS are the ‘worldviews’ which constitute ‘social cognition’ (van Dijk, 1993, p: 258), the ‘representations of aspects of the world which contribute to establishing and maintaining relations of power, domination and exploitation (Fairclough, 2003, p: 218). And the concept of power in CDS is viewed in the Foucauldian sense, who contends that power and domination are embedded and enacted by discourse (Foucault, 1975).

Within CDS, there are different approaches, and five major approaches are identified (Wodak and Meyer, 2016). Based on their linguistic involvement, they can be classified as focusing on only few linguistic devices (e.g., Social Actors Approach) or integrating a broad range of macro-and micro-linguistic, pragmatic and argumentative features (e.g., Discourse-Historical Approach). In this study, Discourse-Historical Approach (hereafter, DHA) is adopted, as the research interest in on the broad range of linguistic devices and strategies for the discursive construction of corporate identity. Among the major approaches, DHA is arguably the most linguistically oriented one (Reisigl and Wodak, 2016), whose proposed analytic framework and tools fit the research goal of this study.

According to Reisigl and Wodak (2016, p: 52), there are three dimensions that DHA focuses on: (1) the specific content or topic(s) of a specific discourse, (2) discursive strategies, and (3) linguistic resources. And they propose five types of discursive strategies, namely, nomination, predication, argumentation, perspectivization, and intensification/mitigation, with each being linguistically realized through a range of linguistic devices.

A note should be made that “strategy” means a more or less intentional plan of (discursive) practices adopted with the aim to achieve a particular linguistic, social, political, or psychology goal. And they are located at different levels of linguistic organization and of different complexity (Wodak, 2015). Also, the categories of discursive strategies and linguistic devices are by no means fixed, but can be adapted to the specific research depending on the data, the research aims, the context, and so on (Reisigl and Wodak, 2016).

Therefore, this study mainly explores three discursive strategies in detail, that is, nomination, predication, and intensification/mitigation strategy. The reason is that this study interests itself more in the most frequent linguistic devices used for Starbucks’ identity construction in its CSR reports, rather than the argumentation schemas (i.e., the topoi *via* which conclusions can be made) and the perspectivization (i.e., whose voices and standpoints are presented). While nomination, predication, and intensification/mitigation strategies can be explored in a quantitative way by using corpus tools to generate results based on the frequency of linguistic devices, argumentation and perspectivization can hardly be explored in the same way.

Since this study focuses on the discursive construction of corporate identity, it takes in the stakeholder theory in the organizational studies and views corporate identity as premised on the relationships the company discursively constructs between itself and its various stakeholders. Therefore, it takes the nomination strategies to examine how the company nominates itself and what stakeholder groups are nominated in the CSR reports, while the predication strategies shed light on what kinds of action define and construct the relationship between the company and its various stakeholders. Intensification/mitigation strategies are explored in combination of nomination and predication to see the degree of certainty, i.e., how strong the company’ voice is, in making the statements of its relationship with the stakeholders.

In addition to the three discursive strategies mentioned above, in this study, another discursive strategy will be included, that is, erasure. It is defined as a form of exclusion or marginalization, particularly in relation to identity categories,’ (Baker and Ellece, 2011). It forms an implicit appraisal pattern, by not mentioning X or using linguistic means to push X into the background. In the daily operations and the interaction with various stakeholders, hardly can any company be exempt from making mistakes and facing challenges, especially for large multinational companies. Therefore, whether companies address the doubts and accusations they harbor and how they respond to their corporate scandals in the CSR reports also constitute their strategies in identity construction and reveals their hidden values and ideologies. Therefore, in this study, with regards to Starbucks’ corporate scandals, investigation will be made on whether or not, and how are they presented in the CSR reports.

## Data and methods

### Corpus of Starbucks’ CSR reports

The corpus under investigation consists of all Starbucks’ CSR reports ever released from the year 2001 to 2020. These reports were downloaded in the pdf form from Starbucks’ official websites, and then converted to plain text (txt.) form. Then, a manual cleaning of the texts was conducted to exclude the following elements in the corpus: 1. captions for any illustrations or photos; 2. tables, diagrams, and figures; 3. the independent report (in the form of a letter) produced by a third party; 4. the section “About the report”; 5. the Mission Statement and Guiding Principles that occur in almost all the reports; and 6. hyperlinks. The total word count of the corpus is 208,444, and the word count for each year’s report is shown in Table 1. The reference corpus in this study is AmE06 Corpus, which is a one-million-word corpus of published general written American English taken from 15 genres of writing.

**Table 1:** Word count for each year’s CSR report.

Size of each CSR report										
Year	2001	2002	2003	2004	2005	2006	2007	2008	2009	2010
Word count	7,710	8,654	15,452	18,634	19,886	29,172	30,634	20,657	5,002	5,247
Year	2011	2012	2013	2014	2015	2016	2017	2018	2019	2020
Word count	5,790	5,298	7,133	3,719	906	5,073	5,989	1810	4,188	7,490

### Corpus-assisted discourse analysis

This study combines quantitative and qualitative methods for the advantages of such an approach (see, e.g., Baker et al., 2008). A quantitative analysis provides a ‘bird’s eye view of the presence of identity (Van de Mieroop, 2007, p: 1122), providing initial insights into the data for further scrutiny, while a qualitative analysis offers more detailed insights into the how identities are constructed in a specific way in the context.

To be more specific, this study is corpus-assisted, using corpus tool WordSmith 5 to process the corpus to generate the quantitative results, based on which a qualitative analysis is made. To be specific, the keyword list is generated, from which significant keywords are manually selected and classified. Next, concordance analysis on the selected keywords is conducted, yielding necessary contextual information about the keywords. In addition, collocation analysis is conducted on the high-frequency keywords, so as to uncover existing patterns of use and reveal what identities are constructed. Moreover, additional steps may follow after the above procedures to shed further light on the discursive strategies and patterns of linguistic resources for identity construction.

## Findings

### The discursive construction of Starbucks

#### Nomination and predication of Starbucks

Starbucks, the company name, serves as the nomination of the company itself in the CSR reports, which appear 2,866 times in total. Collocates that are to the right of the node word ‘Starbucks’ are searched for, and the verbs and nouns are manually coded among the top 30 collocates. The results are shown in Table 2.

**Table 2:** Most frequent verb and noun collocates of ‘Starbucks’.

Categories	Words
Verb collocates	has, is, committed, was, been, will, purchased, purchases, provided, contributed, launched, opened, believes, supports
Noun collocates	partners, coffee, stores, support, communities, commitment, business, growth, experience

First, we will examine the collocate ‘is,’ as its concordances comprise Starbucks’ most explicit identity statements. By a manual examination of all the 179 occurrences, concordances in which ‘Starbucks is’ is followed by nouns are taken out. The linguistic examples can be grouped under the following explicit statements of Starbucks’ corporate identity:

Starbucks as a member of CSR-related organizations

1. Starbucks is also a member of the US Environmental Protection Agency’s (EPA) Green Power Partnership. (2006)
2. That’s why Starbucks is one of the founding members of the Sustainable Coffee Challenge… (2017)

Starbucks as the largest buyer of CSR-related resource

3. Starbucks is the largest buyer of East Timor’s highest-quality Coffee. (2004)
4. Starbucks is the number one purchaser of renewable electricity in its sector on the EPA’s Green Power Partnership National Top 100 list. (2006)

Starbucks as a gathering place and as a good employer

5. Starbucks is a gathering place, a place to connect, a barista offering a cup of coffee with an outstretched hand. (2012)
6. Starbucks is a best place to work. (2013)

We now turn to the other frequent verb collocates of Starbucks, which can give us a clue as to what actions are ascribed to Starbucks as an agent. Based on the actions, we can assign a role to Starbucks. A manual examination of all the concordances of the verb collocates are done, generating the following summary of identity:

Starbucks as an environmental-conscious purchaser:

7. Starbucks purchased considerably more certified organic coffee in fiscal 2005 than in the previous year. (2005)

Starbucks as a CSR projects/initiatives launcher:

8. In fiscal 2002, Starbucks launched two initiatives to improve our recycling rates. (2002)

Starbucks as a provider of funds for CSR causes:

9. Starbucks provided financial support to 42 environmental organizations across North America. (2012)

Starbucks as a supporter of CSR causes/community:

10. As a company, Starbucks supports nonprofit organizations in our communities with cash contributions and product donations. (2004)

After the verb collocates are examined, we now turn to the most frequent noun collocates for Starbucks as pre-nominal modifiers, which can show us what Starbucks is associated with in the CSR reports. Listed below are the identity statements that can be summarized from the noun collocates. Due to limit of space, not all collocates will be illustrated with an example.

Starbucks is about the stores.

Starbucks is about coffee.

Starbucks is about business.

Starbucks is about growth.

Starbucks is about making CSR commitment:

Starbucks is about experience

11. It’s the coffeehouse experience – a third place between work and home – that connects customers to coffee in an inviting, enriching environment that is comfortable and accessible. We call it the Starbucks Experience. (2003)

What is worthy of note is that in (11), a unique term is coined---the Starbucks Experience. This is arguably the most auspicious way for identity construction through nomination. The Starbucks Experience can be understood as a metaphor: Starbucks is a third place for connecting people. A third place is a term frequently mentioned on Starbucks’ website and incorporated in Starbucks’ mission statement and vision. Indeed, this is the identity that Starbucks constructs to set itself apart from the rivals. It has made ongoing and various attempts to brand Starbucks coffeehouses as a “third place” by encouraging patrons to engage in diverse social networks (Rosenbaum et al., 2007). And in the CSR reports, efforts are made to reinforce this identity.

#### Semantic prosody of Starbucks

As the semantic prosody often reveals whether a word is associated with positive or negative evaluations, it is of interest to look into the semantic prosody of ‘Starbucks’ so as to shed some light on its identity construction. A collocate list for ‘Starbucks’ is generated, which results in a total of 994 collocates appearing no less than five times within five slots before and after the node word. Then, a manual examination of all the collocates is conducted, leading to the identification of the adjectives, adverbs, and nouns with evaluative meaning. Among all the collocates, only 48 are identified as having explicit evaluative meanings, among which 33 are associated with positive connotations and 5 with negative connotations, as is shown in Table 3.

**Table 3:** Collocates of ‘Starbucks’ with explicit evaluative meanings.

Semantic prosody	Grammatical category	Collocates
Positive	adjectives	committed, responsible, green, outstanding, positive, sustainable, satisfied, great, good, unique, important, honored, ethical, aggressive
	adverbs	actively, positively, deeply, consistently, responsibly,
	nouns	contributions, success, opportunities, benefits, development, support, change, commitment, impact, progress, improvement, respect, honor, passion
Negative	nouns	challenges, disaster, injury, earthquake, hurricane

From the table, we can see that the semantic prosody of ‘Starbucks’ is overwhelmingly positive, which is in congruence with the Pollyanna Effects found in corporate communication (Hildebrandt and Snyder, 1981). That is, companies tend to put themselves in a positive light while avoiding negative association.

To be noted, the coding of the positive/negative association is dependent on the context in which the collocates appear, instead of merely by the dictionary sense of the word itself. For example, the adjective ‘aggressive’ is often associated with a negative connotation. However, from its specific use in the context, it is decided that this collocate contributes to the positive prosody. As is shown in (12), positive evaluation is assigned to the use of ‘aggressive’ because in the context, it is employed to show Starbucks’ highly ambitious goal, which constructs an identity that is bold, audacious, and forceful in pursuing CSR.

12. Starbucks has set aggressive goals for C.A.F.E. Practices, reflected in the amount of coffee we plan to purchase from participating suppliers. (2005)

In the same manner, the noun collocates which are neutral in themselves (i.e., ‘impact’ and ‘change’) are classified as having positive connotations based on their use in the context. For instance, in (13), ‘impact’ is used in juxtaposition with ‘benefit’ in parallel grammatical structure, so the latter is intended to be interpreted in the same light with the latter. In this way, through deliberate wording, ‘impact’ is invested with positive connotations, which is conducive to the construction of a positive identity for Starbucks. And in (14), ‘change’ is used to testify Starbucks’ constant commitment to CSR in the sense of adopting strong corporate governance practices, which, again, contributes to the construction of a positive corporate identity.

13. Starbucks contributions will have greater impact and provide more benefit to communities around the world. (2006)
14. This change demonstrates Starbucks ongoing commitment to strong corporate governance practices. (2007)

Also worth mentioning is that, there are only five collocates with negative prosody among all the collocates for ‘Starbucks,’ and no adjective or adverb collocates showing negative connotations are identified. Among the noun collocates associated with negative connotations, three detonate external factors beyond the manageability and influence of the company (i.e., ‘hurricane,’ ‘disaster,’ and ‘earthquake’). And the rest detonate things within Starbucks’ control (i.e., ‘challenges’ and ‘injury’), for which proactive efforts are promised.

Also, concordances for ‘challenges’ are manually examined to determine the source of the challenges presented in the CSR reports. And the results indicate that they are presented as being posed or brought about by factors external to the company, with ‘Starbucks’ being constructed as the innocent victim who actively tries its best to cope with these challenges, see (15) and (16). In these cases, an identity of a courageous company who is subject to the challenging external world but takes an active role in living up to the challenges are discursively constructed.

15. The difficult business climate in fiscal 2008 and beyond has brought challenges to Starbucks and the communities we serve. (2008)
16. Through our efforts, we hope to alleviate healthcare challenges for Starbucks and our partners, and all other US companies and employees whose healthcare benefits are threatened. (2007)

With regards to the collocate ‘injury,’ although the negative-meaning-loaded word is used, its mentioning in the sentence is to address stakeholders’ relevant concern explicitly and to showcase Starbucks’ attitude and commitment toward mitigating risks of injury. In this way, Starbucks discursively constructs an identity of a responsible and conscientious company who goes all out to ensure the safety of its stakeholders, as is in (17). And in terms of collocates detonating natural disasters, such as in (18), Starbucks discursively constructs an identity that actively shoulders the responsibility of a global citizen who compassionately provides aides to people in need.

17. We consider partner and customer safety first and foremost as we develop and select Starbucks products and equipment – and strive to “engineer out” as many causes of injury as possible. (2006)
18. In response to the 2011 earthquake and tsunami in Japan, the Starbucks Foundation and Starbucks Coffee Japan gave $1.2 million to the Red Cross for relief and recovery efforts and established a Caring Unites Partners (CUP) fund to help eligible impacted partners in Japan. (2011)

To conclude, the semantic prosody of ‘Starbucks’ is overwhelmingly positive. In the cases of collocates with negative connotations, they are used to construct Starbucks as a responsible, proactive, caring, and conscientious global citizen. And this finding is in congruence with the findings of Fuoli (2018), who found that in the CSR reports, companies use stance markers to construct themselves as committed, honest, and caring corporate citizens.

#### Intensification/mitigation of the voice of Starbucks

Another linguistic resource for nominating Starbucks are the first-person pronouns. As companies are inanimate, it can only speak through its representatives in a collective voice. Therefore, only the plural forms of first-person pronouns (i.e., ‘we,’ ‘us’ and ‘our’) are used as self-referring linguistic devices for the construction of corporate identity. A search of the three devices in Starbucks’ CSR reports shows that there are 3,630 occurrences of ‘we,’ 2,810 of ‘us,’ and 4,908 of ‘our.’

For identity construction, the use of the subject form of first-person plural pronoun ‘we’ is considered to be of primary importance, as it helps construct the company as an active agent in the social world. Moreover, the intensification/mitigation discursive strategy can contribute to the construction of identity by reinforcing or weakening the degree of certainty invested in the statements being made. Therefore, by investigating the collocates of ‘we,’ we can gain some insights on how this discursive strategy is utilized in Starbucks’ CSR reports for identity construction. Among all the resulting collocates, those which serve to intensify or mitigate the voice are identified, with the top six collocates presented in Table 4.

**Table 4:** Top six voice-intensifying/mitigating collocates of ‘we’.

Rank	Collocate	Freq.
1	will	149
2	can	131
3	believe	126
4	know	66
5	must	30
6	recognize	26

On the whole, it is quite revealing that all of the top six collocates serve to intensify rather than mitigate the voice. In order to better understand how are these voice-intensifying devices used in Starbucks’ CSR reports for identity construction, and more importantly, what actions are being intensified, we will explore the collocates for these devices further.

To begin with, verb collocates of ‘will’ are identified. By a careful reading of all concordance lines, it is found that this voice-intensifying device is mainly used to reassure the audience of Starbuck’s strong volition and firm determination to fulfill its CSR agenda. Here, we come across an interesting finding: the most frequent verb following ‘we will’ is ‘continue,’ appearing 40 times in the corpus. The strong commitment made to continuing what Starbucks has been doing is, by itself, an acknowledgment and reinforcement of the righteousness and achievement of Starbucks’ CSR activities, see (19).

19. We will continue to work cooperatively with organizations throughout the world to identify, test and implement the most effective and sustainable energy efficiency initiatives. (2011)

The manual examination of its use in context reveals that the second most frequent collocate ‘can’ also serve to intensify the voice of the self, expressing a relatively strong degree of certainty and emphasizing Starbucks’ capability of helping, improving, and making impacts on the environment and the stakeholders, as in (20). Another prominent voice intensifier on the list is ‘must,’ whose use in Starbucks’ reports is accentuating the strong obligation perceived by Starbucks to take certain actions to fulfill its CSR, see (21).

20. We can use our scale for good, and catalyze change across entire industries so that Starbucks and everyone we touch can endure and thrive. (2012)
21. As we grow, we must focus on engaging with local groups, listening to our neighbors about what’s important to them and determining how Starbucks can best contribute to their neighborhoods. (2004)

Among the top eight collocates, there are three cognitive verbs (i.e., ‘believe,’ ‘know,’ and ‘recognize’), all of which carry a relatively strong degree of certainty. As such, they intensify the corporate voice and construct an identity that is assertive and confident. All of their concordance lines are carefully examined to identify to what end they are used in Starbucks’ CSR reports.

To begin with, of the 110 occurrences of ‘believe,’ three topics are identified. That is, in Starbucks’ CSR reports, ‘believe’ is mainly used to: justify an action (22), emphasize positive impacts (23), and acknowledge the importance of CSR (24). From the examples, we can see that Starbucks uses this voice-intensifying device to constructs an identity that is highly committed and confident in the positive impacts its CSR activities can bring to the stakeholders.

22. We believe the process leads to better results. (2005)
23. We believe the growth of Starbucks has created employment and business opportunities within the specialty coffee industry. (2001)
24. We believe it’s our environmental responsibility to find ways to reduce these emissions. (2004)

The other two voice-intensifying verbs are ‘recognize’ and ‘know,’ whose concordance lines are examined, resulting in the following findings about their usages in Starbucks’ CSR reports. To begin with, over half of the occurrences of ‘know’ convey Starbucks’ explicit acknowledgment of existing challenges and room for improvement, as in (25). Its other usages involve the recognition of the importance of customers’ trust (26) and of farmers’ contribution (27). The use of another voice intensifier ‘recognize’ shows similar patterns, with Starbucks emphasizing its acknowledgment of the imperfections and the need for continuous CSR efforts.

25. Although we are proud of how far we have come since our first report, we know there is still a long way to go. (2010)
26. We know that our customers’ loyalty and trust must be earned. (2007)
27. We know our success as a company is linked to the success of the thousands of farmers who grow our coffee. (2013)

In general, the six most frequent voice adjusting collocates of ‘we’ all serve to intensify instead of mitigate the voice. In other words, they are conducive to the construction of a strongly assertive, confident, and committed corporate identity.

### The discursive construction of stakeholders in the CSR reports

Main stakeholders are identified in the wordlist of the corpus. Altogether, four groups of stakeholders are identified, whose frequencies are presented in Table 5.

**Table 5:** Frequency of the nomination of each stakeholder group.

Stakeholder group	Partners	Farmers	Customers	Suppliers
Frequency	1,585	857	748	531

From the frequency of total occurrences, we can infer the relative weight each stakeholder group bears on Starbucks’ CSR performance. Clearly, the most frequently nominated and thus the most heavily weighted are partners, followed by farmers and customers, and suppliers are least nominated.

#### The discursive construction of partners

According to the frequency of the nomination of partner(s), this stakeholder group is of utmost salience in Starbucks’ CSR reports. Indeed, the fact that Starbucks refers to its employees as partners is a proof that Starbucks recognizes the importance and value of its employees. A collocation analysis is conducted to find out how ‘partner(s)’ is predicated in Starbucks’ CSR reports, with the top six most frequent collocates being presented in Table 6.

**Table 6:** Top six most frequent collocates of ‘partners’.

Collocate	Frequency	Stat.
program	77	4.4
store	72	4.6
support	56	4
benefits	45	5.6
help	42	4.0

As is shown in the table, the most frequent collocate is ‘program,’ and the examination of the concordance lines shows that in Starbucks’ CSR reports, frequent mention is made about the various programs it carries out for partners. The next frequent collocate is ‘store,’ and the examination of its concordance lines show that ‘store partners’ are frequently nominated in Starbucks’ CSR reports. In most of the occurrences, Starbucks explicitly conveys its recognition of the contribution of store partners (28), and acknowledges their significance to Starbucks (29).

28. Our store partners are very innovative when it comes to reducing waste. (2004)
29. Our baristas and their fellow store partners are the face of Starbucks, engaging with our valued customers every day. (2003)

There other three collocates are ‘support,’ ‘benefits,’ and ‘help.’ A close examination of their concordance lines shows that in most cases, partners are the receivers of the support, benefits, and help from Starbucks, as is shown in (30–32). And through voicing the partners in this way, Starbucks constructs for itself an identity of a supportive, helpful, and caring employer.

30. The Thrive Wellness Initiative combines education, communication and participation to help our partners live healthy lives. (2015)
31. We have many programs that encourage and support partners to make a difference in their communities. (2007)
32. Recently, we extended COVID-19 benefits for U.S. par tners, including paid time off to get vaccinated… (2020)

#### The discursive construction of farmers

Due to the specific industry to which Starbucks belongs, farmers who grow coffee beans are arguably the most important stakeholder group in its value chain. Therefore, it comes as no surprise that farmers are frequently voiced in Starbucks CSR reports. The examination of the collocates of ‘farmer(s)’ results in collocates that are identified as serving the predication strategy for the discursive construction of identity, with the top four presented in Table 7.

**Table 7:** Top four frequent collocates that predicate farmers.

Collocate	Frequency	Stat.
support	128	6.2
help	51	5.3
small	44	6.8
loans	39	7.5

An examination of the concordance lines shows that these four collocates are all linked together in voicing and evaluating the farmers. To start with, ‘support’ and ‘help’ both position farmers as the recipients. That is, the farmers are constructed as beneficiaries who receive support and help from Starbucks, as is shown in (33). The help given to the farmers who grow coffee beans for Starbucks is meant both for increasing the coffee bean quality (which is to the advantage of Starbucks) and for providing economic returns for the farmers, as is in (34). By such positioning, the farmers are discursively constructed as relying on Starbucks for their livelihoods.

33. In 2010 alone this support helped nearly 56,000 farmers who grow our coffee in ten countries. (2010)
34. Ultimately, we hope to help farmers increase both coffee quality and yields to help them become more economically stable and more resilient, long-term producers supporting the specialty coffee market. (2013)

The collocate ‘small’ specifically nominates farmers who work on a small coffee farm as important stakeholders for Starbucks. In the CSR reports, these farmers are constructed as being vulnerable and disadvantaged, to whom Starbucks shows much care. By discursively constructing the problems faced by these farmers, Starbucks expresses its steadfast commitment and determination to help solve these problems, as in (35). By voicing the farmers in this way, Starbucks discursively constructs an identity that is the provider of financial help and support for small-scale farmers.

35. We’re steadfastly committed to helping small-scale farmers thrive now and in the future. (2009)

#### The discursive construction of customers

Customers constitutes the third most frequently nominated stakeholder group in Starbucks’ CSR reports. However, a close examination only results in the identification of two collocates that reveal how customers are voiced in the reports, as are shown in Table 8.

**Table 8:** Most Frequent collocates that predicate the customers.

Collocate	Frequency	Stat.
partners	191	8
experience	26	8.4

As is shown in the table, the most frequent collocate is ‘partners,’ which means that the nomination of customers often goes in tandem with the nomination of partners. As such, customers are discursively constructed as sharing common ground and having a close relationship with partners. An investigation of the concordance lines of their co-occurrences shows that they are recognized by Starbucks as a means for the CSR end. To be more specific, Starbucks views customers and partners as the subjects toward whom it has the responsibility to raise their environmental awareness as part of its own CSR calling, as is exemplified in (36).

Why are customers voiced as sharing the same ground as partners in Starbucks’ CSR reports? The answer may be found in (37). That is, in the CSR reports, partners and customers are constructed as constituting the communities that Starbucks serves. In other words, partners are the interface and medium through which Starbucks interacts and builds relationships with the customers.

36. But it also serves as a platform for building awareness among our partners and customers about the responsibility we all share for the environment. (2004)
37. When the holiday season arrives, it heightens the community spirit in our partners and customers. (2007)
38. Earning and maintaining the trust and respect of our more than 145,000 employees – whom we call partners – means improving our customers’ experience and our success as well. (2006)

The collocate ‘experience’ also helps constructs the relationship between customers and partners. In (38), Starbucks equates earning the trust and respect of its partners with improving customers’ experience. In this way, customers are voiced as the service/products receiver of Starbucks, while Starbucks is constructed as a reliable provider of good customer experience.

#### The discursive construction of suppliers

The last stakeholder group voiced in Starbucks’ CSR reports consists of the suppliers. In fact, the total frequency of the nomination of suppliers is significantly lower than that of the other four stakeholder groups, indicating the relatively low importance attached to them. The most frequent collocates predicating the suppliers are presented in Table 9.

**Table 9:** The most frequent collocates predicating the suppliers.

Collocate	Frequency	Stat.
diversity	53	7.1
diverse	37	7.4
preferred	36	9.1

As is shown in the table, ‘diversity’ and its adjective form ‘diverse’ are the top two most frequent collocates that predicate the suppliers. A close examination of their concordance lines shows that in the CSR reports, Starbucks accentuates its commitment to supplier diversity frequently. For instance, in (39), Starbucks discursively constructs its responsibility toward the suppliers as ensuring their diversity, and frames supplier diversity as creating business opportunities and economic impacts.

Another collocate of ‘suppliers’ is ‘preferred.’ In (40) and (41), it is shown that Starbucks evaluates suppliers as being preferable or not based on whether they share Starbucks’ values and meet its requirements, and that the favored ones can receive better contract terms from Starbucks. In this way, Starbucks constructs suppliers as candidates vying for its preferences, and itself as a strict evaluator and selector of the suppliers in its efforts to ensure the coffee beans conform to the CSR values.

39. The commitment we have made to supplier diversity is intended to provide not only opportunities for diverse businesses, but also to create a positive and sustained economic impact on the local communities where these businesses are based. (2006)
40. When doing business with suppliers in the U.S., Starbucks has made a strong commitment to diversity. We select companies that share our core values and meet our key requirements of quality, service, value, stability and sound business practices. (2004)
41. We buy from our preferred suppliers first, paying them higher prices and offering better contract terms. (2003)

### Erasure in Starbucks’ CSR reports

Up to Starbucks’ latest CSR report (of the year 2020), the biggest scandals or problems it has faced include its tax avoidance in United Kingdom reported in 2012, the arrest of two African black men in its store in Philadelphia in 2018, and the ongoing problems of employee unionization throughout the years.

**Figure 1:**
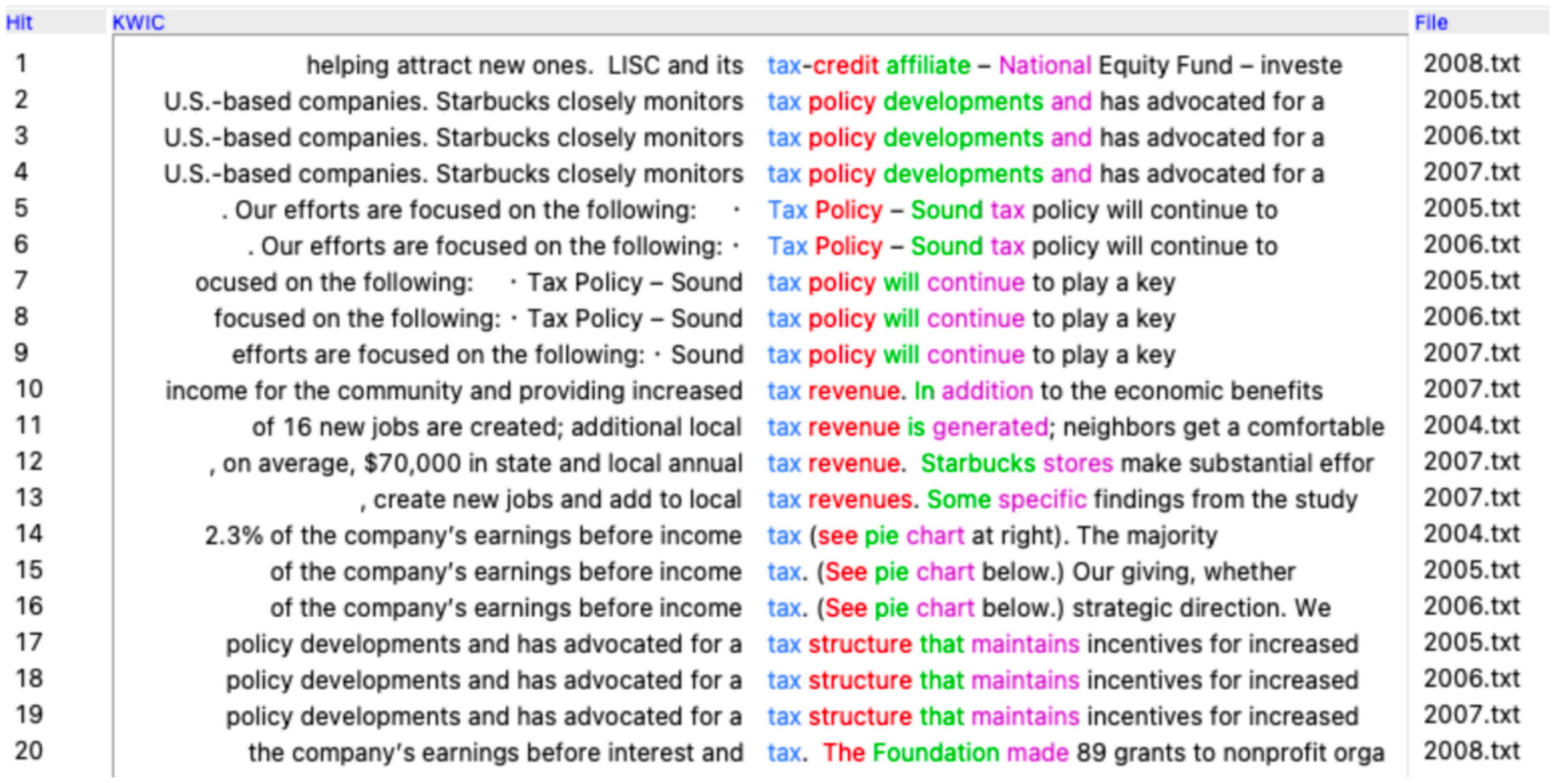
Concordance lines of ‘tax’ in Starbucks’ CSR reports. Reproduced from Antconc (Anthony, 2022).

To begin with, Reuters published a report entitled “Special Report: How Starbucks avoids UK taxes” on October 15, 2012, explaining how Starbucks’ UK stores legally reported no taxable income while generating profits in the United Kingdom since it started operations there. This story has received wide media attention, and Starbucks’ attempts to address the criticism only fuel the criticism from the media, its customers, politicians, and UK tax-paying businesses (Campbell and Helleloid, 2016). A search of the word ‘tax’ in the corpus yields quite revealing results. There are only 20 occurrences of this word in total, and they all come from reports between 2004 and 2008. Figure 1 shows the resulting concordance lines of ‘tax’ in Starbucks’ corpus.

Among the 20 lines, there are several repetitions of the same wording, revealing very formulaic corporate expressions around the topic of tax. A close-reading of the concordance lines show that 12 occurrences come from the same paragraph in the reports of 2005–2007, as is in (42). In its tax policy, transparency and responsibility is not mentioned. Instead, tax policy is only viewed from the light of providing ‘competitiveness’ and ‘incentives for increased productivity.’ Clearly, here tax is not regarded as part of the company’s social and legal responsibility, but serves the profit-seeking end. In this sense, Starbucks’ tax avoidance in UK seems not so much a surprise.

42. Tax Policy – Sound tax policy will continue to play a key role in the competitiveness of U.S.-based companies. Starbucks closely monitors tax policy developments and has advocated for a tax structure that maintains incentives for increased productivity. (2005, 2006, 2007)

**Figure 2:**
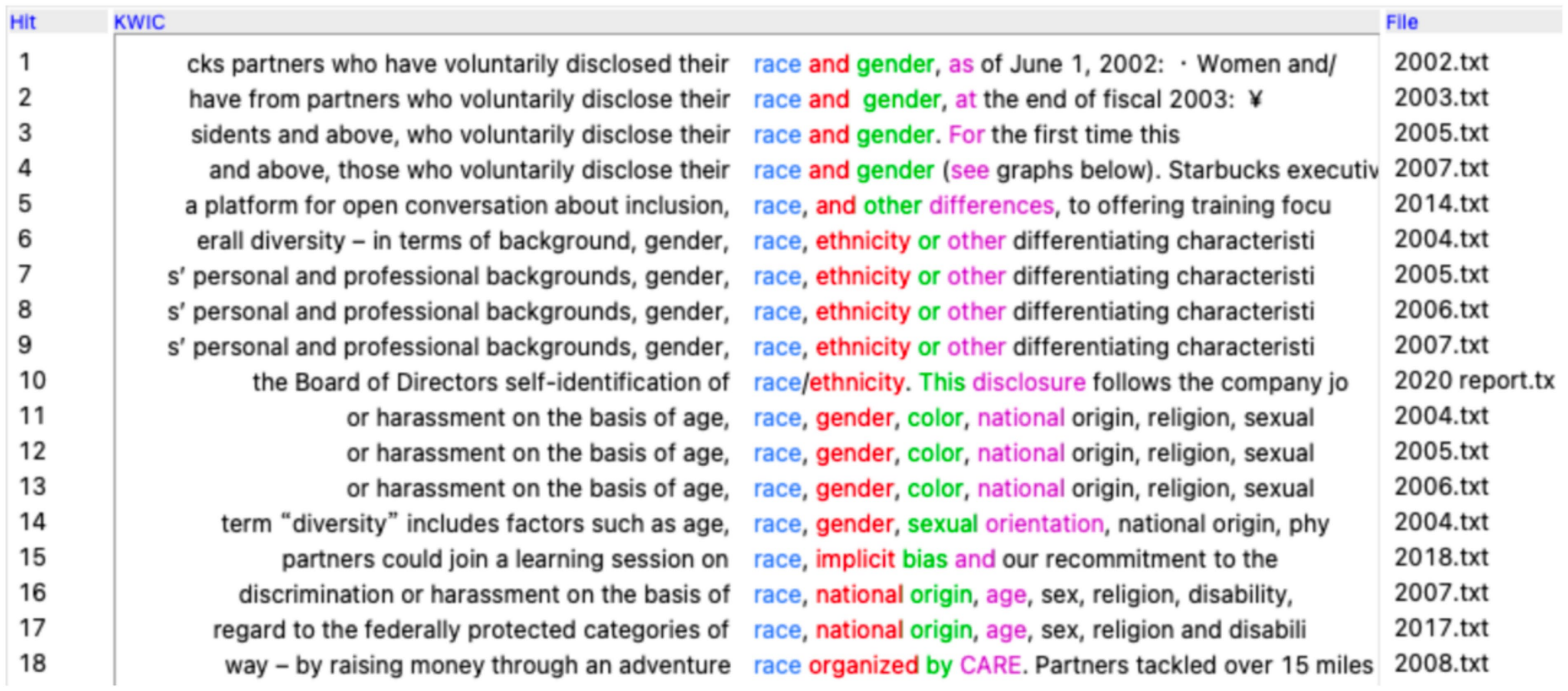
Concordance lines of ‘race’ in Starbucks’ CSR reports. Reproduced from Antconc (Anthony, 2022).

Another scandal of Starbucks involves the arrest of two African black men in its Philadelphian store on April 12, 2018. An employee called the police after the two black men declined to either leave or place an order before the arrival of the colleague they were waiting for. In the wake of the public outrage on this incident, Starbucks responded by calling the arrests “reprehensible” and starting to implement racial bias training for its employees (Karlsen and Scott, 2019). A search of the word ‘race’ in the corpus generates 18 occurrences lines in total, as is shown in Figure 2. However, there is only one occurrence related to the employees’ racial bias against customers, which, understandably, falls in the 2018 report. A close-reading shows that it is mentioned as part of Starbucks’ efforts to implement racial bias training on employee. Other than this single occurrence, all the other occurrences of ‘race’ point to Starbucks’ inclusion of race, gender, age, and so on regarding its partners (employees) as in (43), which constructs an identity of a non-discriminating employer.

43. We are an equal opportunity employer. In addition, we consider all qualified applicants for employment without regard to the federally protected categories of race, national origin, age, sex, religion and disability. (2017)

It is interesting that Starbucks makes no mention about its non-discriminating policy regarding the race of customers in the CSR reports, although it frequently emphasizes its commitment to creating a Third Place for people in its communication to the public. Whether this lack of specific mentioning in the CSR reports can be said to reveal something about the implicit corporate culture is a question open for interpretation.

Another problem related to the stakeholder group of employees is unionization. It is an ongoing problem of Starbucks. Howard Schultz, who founded Starbucks and took the role of CEO several times, have fought against unionization all the along (Durbin, 2022). A search of the word ‘union’ results in 25 concordance lines in total, whose screenshot will not be provided due to the space limit. Among the 25 occurrences, 19 occurrences directly relate to the employee union. In the 2004 report alone, there are 9 occurrences, all referring to the issue of employee union. A close-reading of all occurrences in its co-text provides mush insight on the attitude of Starbucks toward unionization. For instance, in response to the public concern over Starbucks’ conflicts with the partners who wanted to be represented by a union, Starbucks explains that it is the employees’ volition to not unionize, see (44). And in all its CSR reports, Starbucks makes explicit mention of its recognition of employees’ right to unionize 5 times only, in the year between 2003 and 2007, as in (45).

44. The election was to take place, the union voluntarily withdrew its petition, and the election did not take place. We believe that the union realized that the majority of partners who were eligible to vote were not in support of union representation. In addition, the 13 partners in one of our U.S. roasting plants who are currently represented by a union have indicated to that union, and to us, that they no longer want to be represented by the union. (2004)
45. We recognize our partners’ right to organize, and do not take action or retaliate against partners who express their views about unions or who take part in union activity. (2007)

The fact that statements like (45) only appear in the reports between 2003 and 2007 is, in itself, quite revealing about Starbucks’ stance toward employee unionization. Regarding the latest development of this issue, with Starbucks being reported to fire employees who have been involved in unionization efforts, the few mentioning of its acknowledgment of employee unions seems like a glimpse into its long-held attitude.

## Discussion

Following a CDS perspective, this study does not content itself with the analysis of discursive and linguistic elements only. Since discursive practices may have important ideological effects, producing and reproducing unequal power relations between the majority and minority groups through how they use language to represent the world and position people (Fairclough and Wodak, 1997, p: 258). Without awareness of the hidden ideologies behind the discourse, people may buy into the constructed reality unconsciously. Therefore, to reveal the latent ideologies in the discursive construction of corporate identity can help raise peoples’ awareness of the corporate agenda behind, making it possible to call for better CSR undertakings.

### Starbucks’ values and ideologies toward partners

Based on how partners are voiced in Starbucks’ CSR reports, we can see that a two-fold relationship is constructed between Starbucks and its partners. On the one hand, Starbucks relies on its partners’ work, talents, and contributions. On the other hand, as part of its CSR calling, Starbucks has the responsibility to care for both their safety and their wellbeing, providing programs and benefits to support them. Indeed, in such discursive construction, Starbucks constructs an identity that is caring and supportive to the partners, which makes a responsible and reliable employer. The fact that Starbucks calls its employees by the term ‘partners’ is a deliberate effort in constructing a positive corporate identity that hides the power relation inherent in corporate capitalism.

In such discursive construction, Starbucks skillfully obscures the capitalistic ideology that employees’ value as labor force is to be maximumly exploited. And the responsibility to the employees, for instance, providing safe work environment and healthcare, is premised on employees’ value creation for Starbucks. Besides, as have been shown, in the CSR reports, Starbucks makes little recognition of the partners’ right to unionize. This somehow goes against an identity of employer who respect the rights of the employees. And the power relation between them is anything but equal, with Starbucks dominating the employees.

### Starbucks’ values and ideologies toward farmers

In Starbucks’ CSR reports, farmers are voiced and evaluated as being vulnerable and thus highly dependent on Starbucks for their livelihoods. The ideologies behind such discursive construction are also capitalistic in nature, as farmers are positioned as being inferior and thus having little power. Starbucks discursively constructs the problem faced by the farmers as something beyond their own means. In this way, it constructs itself as the solution to the problem. In essence, this is an unequal relationship, with Starbucks having power dominance over the farmers. And to mask such unequal power relations, Starbucks discursively and rhetorically constructs a mutually dependent relationship between the farmers and the company.

On the other hand, in the discursive construction of its identity as the provider of help to farmers, Starbucks obscures the fact that such help also benefits itself. In this sense, Starbucks positions itself in a somewhat altruistic way. That is, it is helping the poor and vulnerable farmers because it is a responsible citizen who cares for the farmers in need.

However, the goal of Starbucks’ transaction with the farmers is to get the coffee beans to make its products so as to earn profits. And in fact, Starbucks cannot survive without buying the beans from the farmers. It just happens that the coffee bean farmers are in the under-developed areas suffering from poverty, so Starbucks can constructs its action of buying from them as heling them, shielding the fact that this transaction serves its own profit-making end.

### Starbucks’ values and ideologies toward customers

In the context of the business world, customers can be said to be one of the most important stakeholder groups (the other being investors) for any company. And the relationship between a company and its customers is based on exchange, i.e., providing goods/services in exchange for profits. However, in Starbucks’ CSR reports, customers are not voiced as the provider of economic returns for Starbucks, but as the stakeholder group to which it has the responsibility to serve by providing consistent and satisfactory customer experience. As such, Starbucks obscures the exchanging nature of its relationship with the customers. In this sense, customers are positioned as the taker while Starbucks is positioned as the giver, constituting a corporate identity that is responsible and sensitive to customers’ needs.

However, Starbucks’ erasure of the issues of employees’ racial bias toward the customers may cast some doubt on its commitments to create a third place that is inviting.

### Starbucks’ values and ideologies toward suppliers

Suppliers are an important link in the value chain for a company. However, the demand for suppliers is relatively stable and fixed, whereas the supply of suppliers obviously exceeds the demand. As such, the nature of the relationship between Starbucks and the suppliers is also unequal, with Starbucks the selector and evaluator having power dominance over the suppliers who are being evaluated without much agency.

On the other hand, the discursive construction of a corporate identity that is strongly committed to supplier diversity, to some extent, masks the fact that in essence, Starbucks will only choose and include diverse suppliers to its own advantage. Besides, Starbucks does not mention in the CSR reports that the diversity of suppliers actually contributes to the diversity of Starbucks’ products (e.g., coffee beans from different farms in different regions, as well as tea and cocoa bean), adding to its strengths and competitiveness.

## Conclusion

This study conducts a corpus-assisted critical discourse analysis on Starbucks’ CSR reports to shed light on how Starbucks utilizes the discursive strategies and linguistic resources to construct its corporate identity in the CSR dimension. Through its positioning of the various stakeholders, Starbucks constructs itself as a supportive care-taker of the partners, committed provider of good customer experience, powerful helper of the poor farmers, and CSR-conscious selector of suppliers. Moreover, these identities are further reinforced by Starbucks’ use of intensification strategy to emphasize its CSR commitments, contributing to a proactive CSR stance.

However, viewing Starbucks’ identity construction from a critical perspective, we can be aware of the hidden corporate capitalism behind its discourse. Starbucks’ relationships with the main stakeholder groups are all based on its corporate capitalistic nature, serving the profit-seeking end. In other words, its very own survival relies on the transactions with these stakeholder groups. Despite its discursive efforts to construct a positive identity, this study finds that it ‘erased’ the corporate scandals or problems about tax avoidance, employees’ racial bias, and its own attitude toward unionizations. Besides, on a more general level, corporate capitalism entails the maximization of business growth and economic development, which necessarily creates the tension between companies and the environment as well as society at large (Kazmi et al., 2016).

Therefore, we need to take a more neutral and objective stance toward companies’ discursive construction of a socially responsible identity. If the whole society can be more aware of the companies’ roles and stances in CSR, as well as the hidden values behind in the CSR communication, companies may be prompted and pressured to take CSR more seriously and engage in more CSR activities that will truly benefit the environment and the society.

The limitations of this study lie in the relatively small size of corpus and the lack of a diachronic perspective on the features of identity construction in different time periods. Also, due to the space limit, the discussion of the hidden values behind the discourse unfortunately may not go as deep as would be expected from a critical perspective. Future studies may consider investigating other corporate discourse (e.g., corporate annual reports, press releases, corporate websites, social network accounts). Second, comparative studies can be conducted to see whether there are industry-specific and/or country-specific features in companies’ discursive construction of the corporate identity. Also, studies can take a diachronic view to find out to what degree does companies’ identity construction vary in different time periods.

## Data availability statement

The raw data supporting the conclusions of this article will be made available by the authors, without undue reservation.

## Author contributions

XL contributed to the conception and design of the study, the collection and preparation of the corpus data, the analysis of the data, and the writing of the manuscript.

## Conflict of interest

The author declares that the research was conducted in the absence of any commercial or financial relationships that could be construed as a potential conflict of interest.

## Publisher’s note

All claims expressed in this article are solely those of the authors and do not necessarily represent those of their affiliated organizations, or those of the publisher, the editors and the reviewers. Any product that may be evaluated in this article, or claim that may be made by its manufacturer, is not guaranteed or endorsed by the publisher.
